# Distributed and Modular CAN-Based Architecture for Hardware Control and Sensor Data Integration

**DOI:** 10.3390/s17051013

**Published:** 2017-05-03

**Authors:** Diego P. Losada, Joaquín L. Fernández, Enrique Paz, Rafael Sanz

**Affiliations:** 1 Robotics and Control Unit, AIMEN Technology Centre, Porriño 36410, Spain; 2 Department of System Engineering and Automation, University of Vigo, Vigo 36310, Spain; joaquin@uvigo.es (J.L.F.); epaz@uvigo.es (E.P.); rsanz@uvigo.es (R.S.)

**Keywords:** mobile robots, distributed control, CAN bus, software agents, software components

## Abstract

In this article, we present a CAN-based (Controller Area Network) distributed system to integrate sensors, actuators and hardware controllers in a mobile robot platform. With this work, we provide a robust, simple, flexible and open system to make hardware elements or subsystems communicate, that can be applied to different robots or mobile platforms. Hardware modules can be connected to or disconnected from the CAN bus while the system is working. It has been tested in our mobile robot Rato, based on a RWI (Real World Interface) mobile platform, to replace the old sensor and motor controllers. It has also been used in the design of two new robots: BellBot and WatchBot. Currently, our hardware integration architecture supports different sensors, actuators and control subsystems, such as motor controllers and inertial measurement units. The integration architecture was tested and compared with other solutions through a performance analysis of relevant parameters such as transmission efficiency and bandwidth usage. The results conclude that the proposed solution implements a lightweight communication protocol for mobile robot applications that avoids transmission delays and overhead.

## 1. Introduction

Mobile robots need very reliable navigation capabilities to operate for long periods of time autonomously. The navigation system is one of the main parts of a control architecture, but its performance will always depend on the information quality gathered from the sensors and provided to the actuators. Therefore, to design a reliable application based on mobile robots it is necessary to have a robust sensor integration and hardware control system.

The information provided by some sensors can be critical and must be managed according to the control process that makes use of it. Obstacle avoidance, localization or map construction are critical tasks while head pan-tilt movement or voice synthesizer are usually non-critical. The current wide range of mobile robot architectures leads to different approaches in the organization of these tasks, and also in the information management in the lower layers where the hardware is connected. Most architectures split the different tasks in several layers. In the same control system, more than one module can make use of the same information; it can also be used in different levels. For example, one module can use raw data from a sensor and another one can use it filtered through another module that synchronizes it with other sensor information. This complexity in the information handling could lead to a high computational load if the hardware layer is not well integrated into the control architecture.

Over the last decades, as a result of the efforts made in the field of mobile robots, different solutions and implementations of robot control architectures have been proposed. Nowadays, the main research topics in this field are focusing on robotics development environments, designed to create applications based on mobile robots. Some of these environments contain a control architecture and tools to develop new modules, to improve control algorithms or to debug the current system [1], while others do not implement a specific control architecture. Instead, they have the necessary tools to build it, including communication systems, configuration and management modules, graphic interfaces and programming libraries. These architectures and environments generally tend to ignore the hardware development. They are centered only on the software, implementing the interface with the hardware devices using a communication library over standard interfaces such as Ethernet, serial or USB (Universal Serial Bus).

The aim of this work is to create a modular architecture for distributed hardware control and sensor data integration in the field of mobile robots. We integrate it as a new tool into RIDE (Robotics Integrated Development Environment) [2] but it can be easily adapted to other environments such as ROS (Robot Operating System) [3].

Some previous works in the field of robotics deal with the same problem proposed in this article. In [4], a modular, distributed and scalable architecture for spacecraft avionics is presented. The solution proposed by the authors relies on the use of autonomous distributed units, in charge of specific tasks and interconnected with a fiber optics communication bus. Every distributed unit follows a similar design concept using common board dimensions, enclosures and also some power converter design. In [5], another distributed hardware architecture is presented for spacecraft/robotics vehicles. The authors present a new technology based on REUs (Remote Engineering Units) developed to work autonomously and communicate through an $I^{2}C$ bus.

These previous approaches are centered on the creation of reusable units to improve reliability and robustness, decreasing the development cost at the same time. This allows to avoid testing and debugging complex hardware designs by dividing the work between distributed hardware modules. The solution proposed in this work consists of a set of distributed hardware modules to integrate sensor data, manage the actuators and also perform distributed control.

The main difference between the previously mentioned works and our proposed solution is the embedded software of every distributed hardware module. Every hardware module is based on a microcontroller unit and the software is developed under an agent–component design paradigm.

For several years, researchers in the field of computer engineering and robotics have been exploring configurations based on software agents and components. The results are control architectures such as DCF (Distributed Control Framework) [6], where a deep analysis of distributed control software agent development is done, [7] or [8] with a component-oriented design for distributed real-time control systems. Component software development has also been applied to industrial environments [9], proving the robustness of systems obtained with this design paradigm. Even though a lot of solutions have been developed, few of them are related to embedded programming, and most of them are distributed but implemented in general purpose computers. RoboCAN (Robot CAN bus) allows both the agent and component software engineering to be applied in the design of distributed hardware modules with a microcontroller unit.

Communications with the distributed modules are implemented using a CAN fieldbus [10] with a lightweight application layer. Different factors have been considered for this choice. CAN bus protocols are widely employed in vehicles and other applications that include a considerable number of interconnected devices [11]. Another reason to select this bus is the remarkably high number of integrated circuits in the market with the CAN interface. They are also accessible at relatively low prices, in part because of their use in the automotive industry. The CAN bus has been successfully used in several engineering domains, including critical control systems as is presented in [12].

The rest of the paper is organized as follows: the next section introduces the CAN-based communications. The robot control architecture is presented in Section 3, continuing with the RoboCAN tool description in Section 4. Section 5 includes the slave modules and Section 6 the RoboCAN server. In Section 7, the hardware technology is described. The article finishes with an analysis in Section 8 and conclusions in Section 9.

## 2. CAN-Based Communications

CAN is a serial communication bus originally developed by the German company Bosch for the automotive industry, envisaged to replace the existing complex wiring harness with a two-wire bus [13]. It has a high immunity to electrical interferences, making it more suitable to use with big machines than other solutions such as the ones reviewed in [14] that include $I^{2}C$. It has also self-diagnose and data error recovery capabilities. These features explain the CAN bus popularity in a wide variety of industrial domains including automotive, marine, medical, manufacturing and aerospace.

The CAN standard protocol defines the first two layers in the OSI model, corresponding with the physical and the data interchange layers [15]. As the CAN standard protocol does not define the application layer, many protocols based on CAN have been developed. Among the different CAN communication protocols are DeviceNet, Smart Distributed System (SDS), CAN Kingdom, CAN Application Layer (CAL) and CANOpen. There are other protocols such as CANaerospace [16], designed for avionics systems, which is used as a backbone network for flight state sensors and navigation systems. The CAN communications have been also used before in different real-time implementations, such as rtfCAN [17] and SCoCAN [18], where a hybrid event and time triggered protocol with separated time frames in transmission are used. Several mobile robots use CAN communications: in [19], a time triggered protocol (FTT-CAN) is presented and in [20] a low level and real-time gateway between the reactive and deliberative levels of the control architecture is used. On the other hand, the protocol (SC-CAN) is an extension of the high level Ethernet protocol. Another approach was presented in [21], where the authors have a similar distributed sensor and actuators integration approach, but with modular units for distributed processing. In some cases, such as [22], hybrid communication structures that take into account hybrid navigation architectures are used. However, they propose quite complex communication frameworks for real-time distributed systems.

In the distributed control schema of RoboCAN, it is not necessary to have heavy, complex or restricted protocols. It is possible to implement a lightweight solution for mobile robot applications avoiding transmission delays and overhead. To this end, RoboCAN includes an application layer protocol with device addressing, flow control and transportation of large data blocks in several frames.

## 3. Robot Control Architecture

Our research addresses mobile robot applications that include control, supervision and collaboration of several mobile robots. More specifically, it is based on a set of robots working under real-time restrictions in dynamic environments, which are usually non-fully specified. The robots must be able to carry out remotely requested plans and react to unexpected events. The design and implementation of every application relies on RIDE [2].

RIDE is a set of tools and a multirobot and multiuser control architecture to design, develop, run and debug applications based on mobile robots. The control architecture can be divided in two blocks: the individual control architecture that manages each robot, which runs into the on-board computer, and the multirobot control architecture executed in a remote server. This work focuses on the first block, the individual control architecture that will be briefly described in this section.

The architecture is based on a modular and distributed software system with centralized communications. Even though the different modules are organized in four sets, they can be mapped in the three-layer architecture popularized by Bonasso et al. [23]. The hardware servers and control set implement the functional layer, while RoboGraph *Dispatch* implements the executive and planning layer. Finally, the architecture includes a set of processes to interact with the users and to connect to the Central Server using a wireless communication system [24].

The navigation platform is based on CARMEN (The Carnegie Mellon Navigation Toolkit) [1] and some modules, such as *localize*, *navigate* and base hardware server interfaces, remain basically the same with new added capabilities such as an infrared localization system [25]. Unlike CARMEN, motion control is divided into high-level (strategic) planning and a lower level (tactical) collision avoidance using the Beam method [26]. Other modules have been added to enhance the architecture and cover some needs and weaknesses.

The different architecture layers and the sets of modules are briefly described in the next subsections.

### 3.1. Hardware Server Layer

The modules included in this layer lead the hardware interaction, providing an abstract set of sensor and actuator interfaces that isolate the control methods from the hardware details. Some of these devices are used in navigation, i.e., laser and sonar, while others are specific for the application, such as the robot head or the sound and speech systems.

### 3.2. Control Layer

The modules in this layer integrate sensor and motion information to provide improved sensor odometry, basic navigation capabilities (localization, path planning and reactive control) and basic application specific functions. The behavior of the robot depends on the internal evolution of every control module as well as on the synchronization between them.

### 3.3. Executive Layer

All the modules in this layer belong to the RoboGraph tool. It includes two modules: RoboGraph *GUI* (Graphic User Interface), used only for applications development, and RoboGraph *Dispatch*. RoboGraph is a tool to create complex tasks using Petri nets. The *GUI* is the programming IDE, used to create and debug the Petri nets that coordinate the evolution of different control modules. These tasks are stored as Petri nets in XML files, that are loaded and executed by the *Dispatch* in running time on demand. The execution requests mainly come from the control modules or user graphical interfaces executed in the upper layers of the multirobot architecture.

### 3.4. Interface Layer

There are several modules in this layer for application developers to debug and trace the control and hardware server modules. However, there is only one interface module on board that allows the application end user to interact with the robot. *RobotWeb* is the module that exchanges information between the robot and the Central Server station where the multirobot control system runs.

Most of the applications developed with RIDE are designed to have multirobot and multiuser capabilities. This makes it necessary to take control over the available resources, the environment where the robots operate and also the synchronization and optimization in the execution of complex tasks for every robot. The Central Server is in charge of covering all these needs, executing a set of control modules to manage the traffic, the task execution and the interaction with the environment, including doors, elevators, sensors, alarms and so forth.

**Figure 1:**
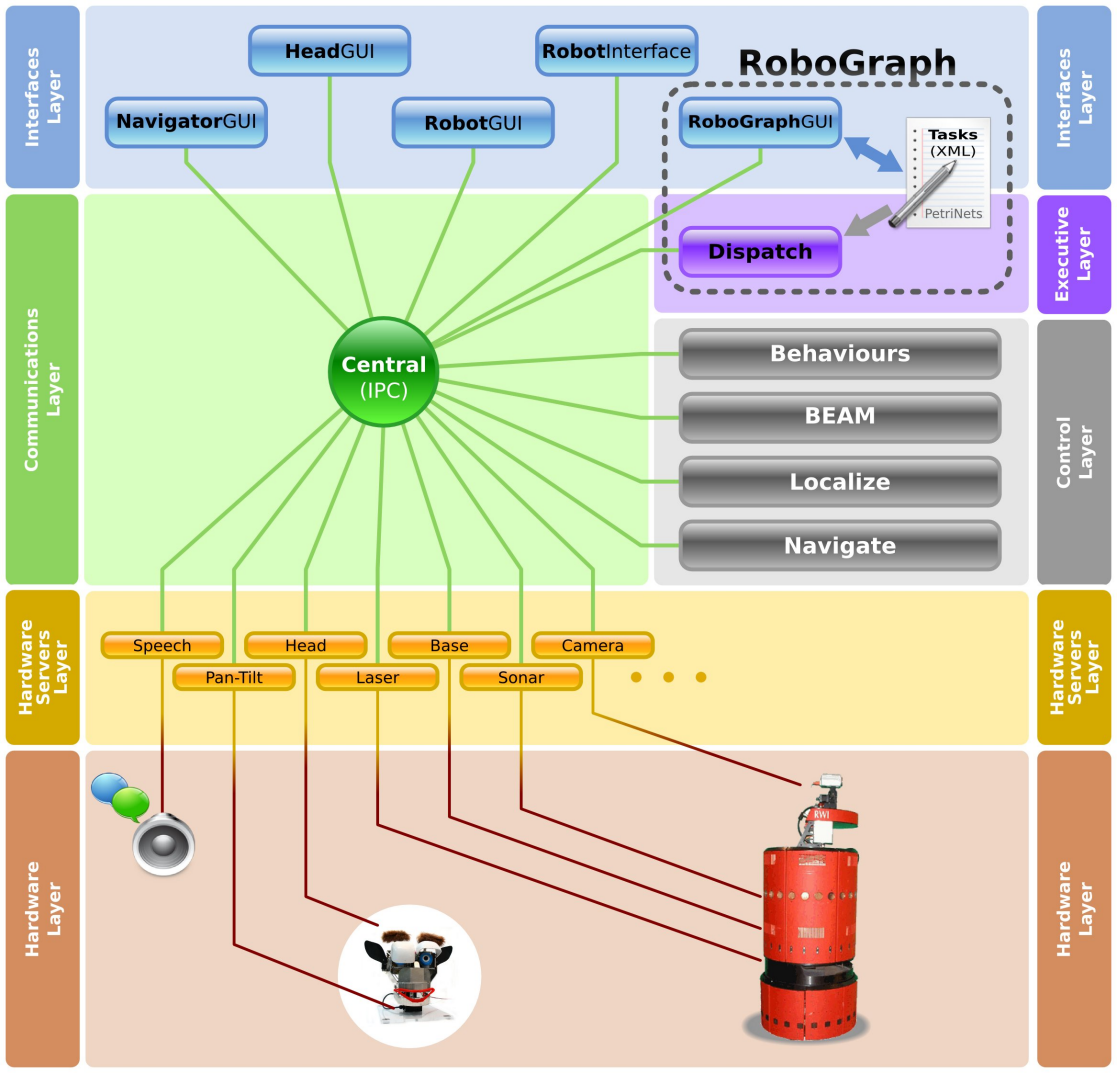
RIDE (Robotics Integrated Development Environment) robot control architecture represented by modules and layers.

The RoboCAN development affects only the two lowest layers depicted in Figure 1, the hardware layer and the hardware servers layer. The main idea is to design a tool to integrate sensors and actuators used in robots in a simple way, to improve the reliability of the system and to increase the robustness.

## 4. RoboCAN Tool

In this section, the RoboCAN integration and control tool is presented. In Section 4.1, the changes introduced in the robot control architecture are described, and the way the hardware subarchitecture is integrated inside RIDE is analyzed. In Section 4.2, the CAN bus protocol, used to make the hardware devices communicate with the master module, is depicted.

### 4.1. Architecture

In the previous section, the robot control architecture depicted in Figure 1 was described. The RoboCAN tool is located in the two lowest level layers, the hardware servers and the hardware, so the use of this tool extends the RIDE capabilities, including the hardware as a part of the control architecture.

**Figure 2:**
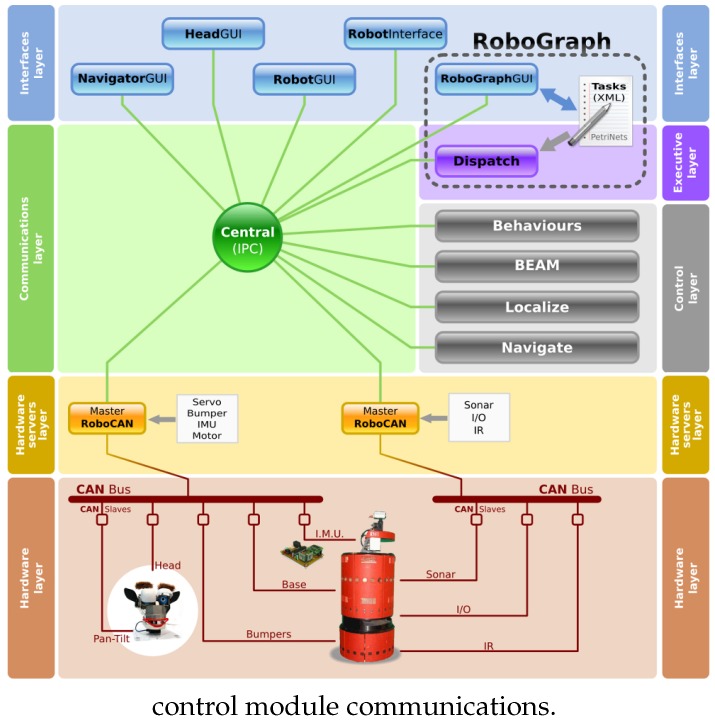
Robot architecture. CAN-based sensor–actuator and IPC (Inter Process Communication).

Figure 2 shows the new control system arrangement, where a unique module placed on the hardware servers layer is represented, the RoboCAN server. The hardware subarchitecture is designed as a single master communication system, even though the CAN is a multimaster bus. It is possible to use more than one RoboCAN server over different and isolated CAN buses, as it is represented in Figure 2. Every RoboCAN server manages all the distributed hardware devices attached to the communication bus. The servers have to implement the same interfaces over the IPC (Inter Process Communication) [27] process used by the old hardware servers (Figure 1), in order to avoid any change in the upper layers modules. The use of RoboCAN is fully compatible with the previous system. This is very important since not all the hardware devices can be integrated with a low bandwidth communication system. This is the case for cameras, laser scanners, and other kinds of sensors.

The field buses with the distributed slave modules are included in the hardware layer. Each one of these modules is based on a microcontroller unit (MCU) running a program that might include control capabilities. They are intended to implement the specific sensor and actuator interfaces, depending on the hardware connected to the module. With this new set of distributed devices, RIDE integrates the hardware in the application design process, resulting in a more compact and robust robot control architecture. The robustness improvement is related with the reduction of the number of ports and links between the two lowest layers. In the previous architecture, a link for every hardware device was necessary, incrementing the sources of potential malfunctions. This problem is now avoided using a two-wired communication bus with a single link with the on-board robot computer.

### 4.2. Communication Protocol

As mentioned before, the main goals underlying the design of RoboCAN tool is to have a centralized, lightweight, scalable and open system to integrate sensors and actuators into the control architecture. It is open to any kind of sensor, actuator and distributed control unit, providing the programmer with a set of basic functions to include new drivers for new devices. For such reasons, RoboCAN contains a very simple protocol that matches the next design requirements:

- Hot plug device connection. The protocol allows to connect and to disconnect distributed modules while the system is running. These operations are detected by the RoboCAN server in order to act accordingly, since some modules could be critical while others could not.
- Remote configuration. Distributed modules can be configured remotely by the RoboCAN server, changing the control method, the behavior or the information management, depending on the kind of system connected.
- Extensible. It is easy to add new modules and drivers to the RoboCAN server in order to introduce new sensors, actuators or control modules in the architecture.
- Robust. The CAN nodes must keep communicating even when some sensor, actuator or distributed unit fails, letting the RoboCAN server program decide whether those failing elements are critical or whether it is feasible to keep the robot operating.

The master process handles the connection, disconnection, configuration and working mode of the different slave nodes, loading the corresponding drivers. This is done in a similar way as USB devices can be plugged into and unplugged from a computer. Modules can be connected at any time and in any order. When plugged, every module starts sending a watchdog frame periodically. The server realizes then that there are new connected modules and sends query frames in order to know what has been attached to load the corresponding driver, configure the correct parameters and set the working mode that defines the information and commands exchanged.

Every slave module uses an unique identifier (ID) to publish frames, which can be any even number. It receives frames with ID + 1, the next odd number, and also receives the broadcast frames with ID 0x01. The server handles all the even ID frames and publishes odd ID frames.

## 5. RoboCAN Slave Modules

**Figure 3:**
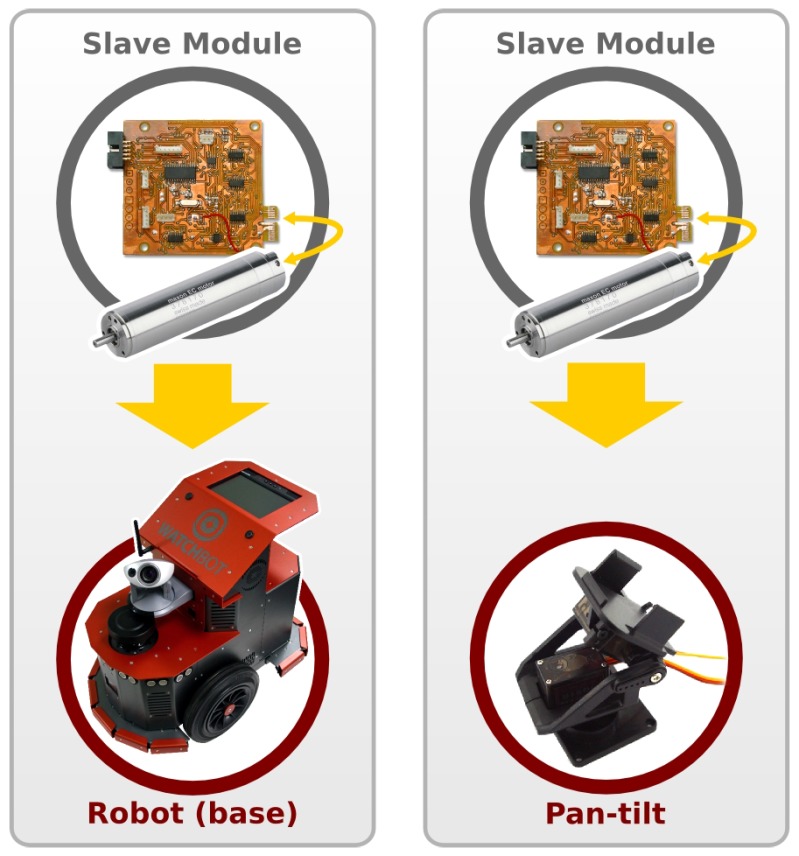
The same module (motor controller) used in two different hardware systems. (**left**) Mobile platform; (**right**) Pan-tilt unit.

A RoboCAN slave module is a set of hardware devices and software elements. The hardware is based on a microcontroller unit with peripheral communication interfaces while the software elements can be components or agents. The main difference between this new implementation method and other works previously presented, such as [5], lies in the use of general purpose distributed software instead of general purpose hardware. Every RoboCAN module is intended to integrate a short number of hardware elements to be totally reusable, so it could be easily installed in different machines or used in different systems. For example, a motor control module can be part of a robot base together with other equal module, or it can be also used in a pan-tilt unit (Figure 3). The functions assigned to the module are exactly the same in both systems. This way, we increase the flexibility, adaptability and scalability creating hardware devices, avoiding possible obsolescences by allowing modifications or even the replacement of some modules.

From the point of view of the control architecture, the distributed modules contain two types of embedded software elements. The first type includes the interfaces to gather the sensor data from the hardware or to activate, and also manage, the different states of the actuators. These elements are implemented as software components, so only one software component for every kind of sensor or actuator is necessary in the slave module. For example, a slave module that controls eight sonar sensors and ten bumpers only needs to include two different components: the sonar and the bumper. The second type of software element is agents. Agents are pieces of control programs that use the information gathered by the sensors and can change the state of the actuators. These agents represent the RoboCAN subarchitecture distributed control capability. Some examples of control functions already implemented are the motor controllers (PID and Predictive PID), the Kalman filter implemented in the IMU (Inertial Measurement Unit) and also the different firing sequences used to manage a set of sonar sensors controlled with more than one slave module.

The combination of software components and microcontrollers allows designing simple and robust distributed modules through the use of different MCU, which can execute the software components developed for a specific hardware architecture. RoboCAN includes components created for 8- and 16-bit architectures, but most of them were developed to be executed in the 16-bit 30F PICs (Peripheral Interface Controller) family.

Component software development is usually related with an operating system that provides an abstraction layer over the peripherals. Programming embedded microcontrollers without an operating system forces the use of abstraction tables to keep the software independent from the hardware used, allowing to choose the best MCU model for every new module design.

**Figure 4:**
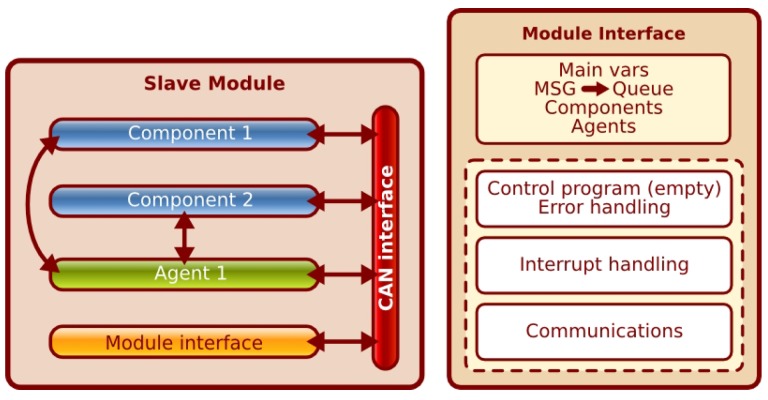
Software blocks and structure of a slave distributed module.

The left side of Figure 4 shows the different software blocks involved in a slave module. These blocks are the components, the agents, the module hardware abstraction layer and the communications interface. Both components and agents were previously described. The communications interface is basically a library to manage the CAN buffers using queues to receive and send data frames. The hardware abstraction layer is the specific software created for every module. It is related to the microcontroller model to define the proper processor configuration, such as communication interfaces, instruction time and peripherals. This software manages the components throughout interruptions. The RoboCAN distributed software elements are intended to be driven with both internal and external interruption sources. The external interruptions sources are the CAN communications and some inputs used for a particular sensor or actuator. The internal ones are, in most cases, timers controlling the real-time execution of every element software cycle. It is also possible to use interruptions from some peripheral interface, allowing more flexibility to handle the software elements. The right side of Figure 4 represents the module interface structure. This structure contains a set of global variables to configure the hardware, the communications queue and also contains two lists with components and agents included into the module. The module functions are the error handling, some specific hardware control function if necessary (usually empty), the interrupt handling and the communications management.

## 6. RoboCAN Server

The RoboCAN server is a process executed in the on-board robot computer. It integrates the distributed hardware modules into the control architecture. This process also includes the communication field bus master node, which handles the connection procedure of the different slave nodes, configuring each of them if necessary (changing the default working parameters).

The server works between two information levels inside the control architecture. Therefore, it uses two communication systems to connect the distributed hardware modules with the upper control layers. The first one is the CAN-based communication system, included as part of the RoboCAN tool to develop hardware control and sensor data integration modules. The second one is IPC, described later in this section. From the point of view of the data management, the server integrates the information gathered by different sensor nodes attached to the CAN bus, and provides it to the software modules in the upper levels of control. This information is shared using the IPC publish/subscribe paradigm. In the other direction, the server takes the information from upper layers and converts it in lower level information suitable for the distributed hardware modules.

**Figure 5:**
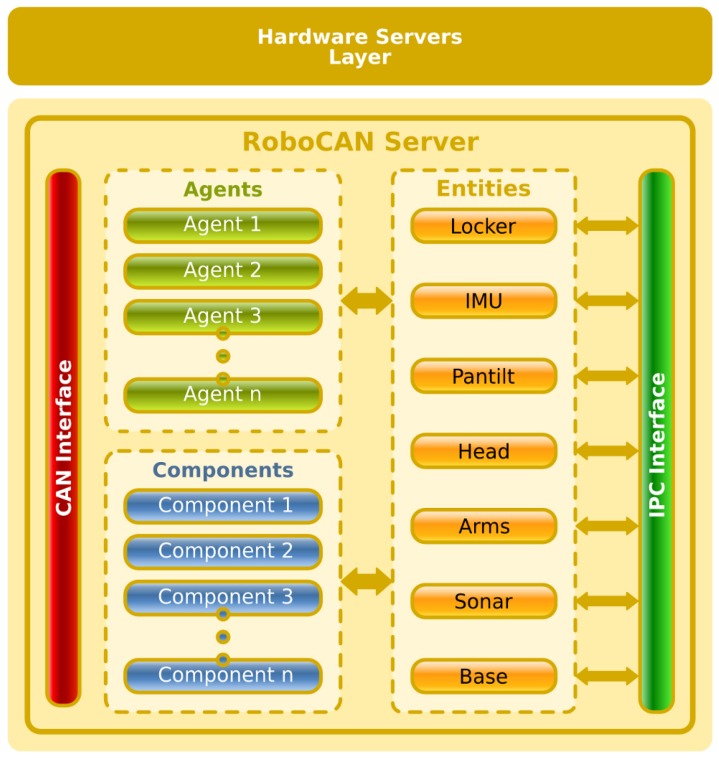
RoboCAN server structure.

The server structure is depicted in Figure 5, showing how the data is managed between the two information levels. The interface with the CAN bus is represented in the left part of Figure 5. This interface is used to communicate the master node with agents and components running in the distributed slave nodes. The interface with IPC is represented in the right part of Figure 5. This interface integrates the low level information into the upper layers and translates the high level command messages, coming from the upper layers, into a set of CAN bus frames. The server handles the distributed software using drivers for every kind of agent or component. It handles the IPC integration with data structures, represented in Figure 5 as *entities*, which are gateways between the drivers and the high level communication interface.

RoboCAN tool can be easily integrated in other control architectures, such as ROS, by changing the high level communication interface and adapting the *entities* to the new information exchanged. To integrate a RoboCAN robot into ROS, the only RoboCAN module from Figure 2 that needs to be changed is the *Master RoboCAN* but all the slave modules will remain unchanged. The communication system used in ROS is based on a publish/subscribe paradigm such as IPC. Therefore, the changes to integrate the *Master RoboCAN* module are limited to a few *entities* that use the IPC interface (see Figure 5).

### 6.1. CAN Communications

**Figure 6:**
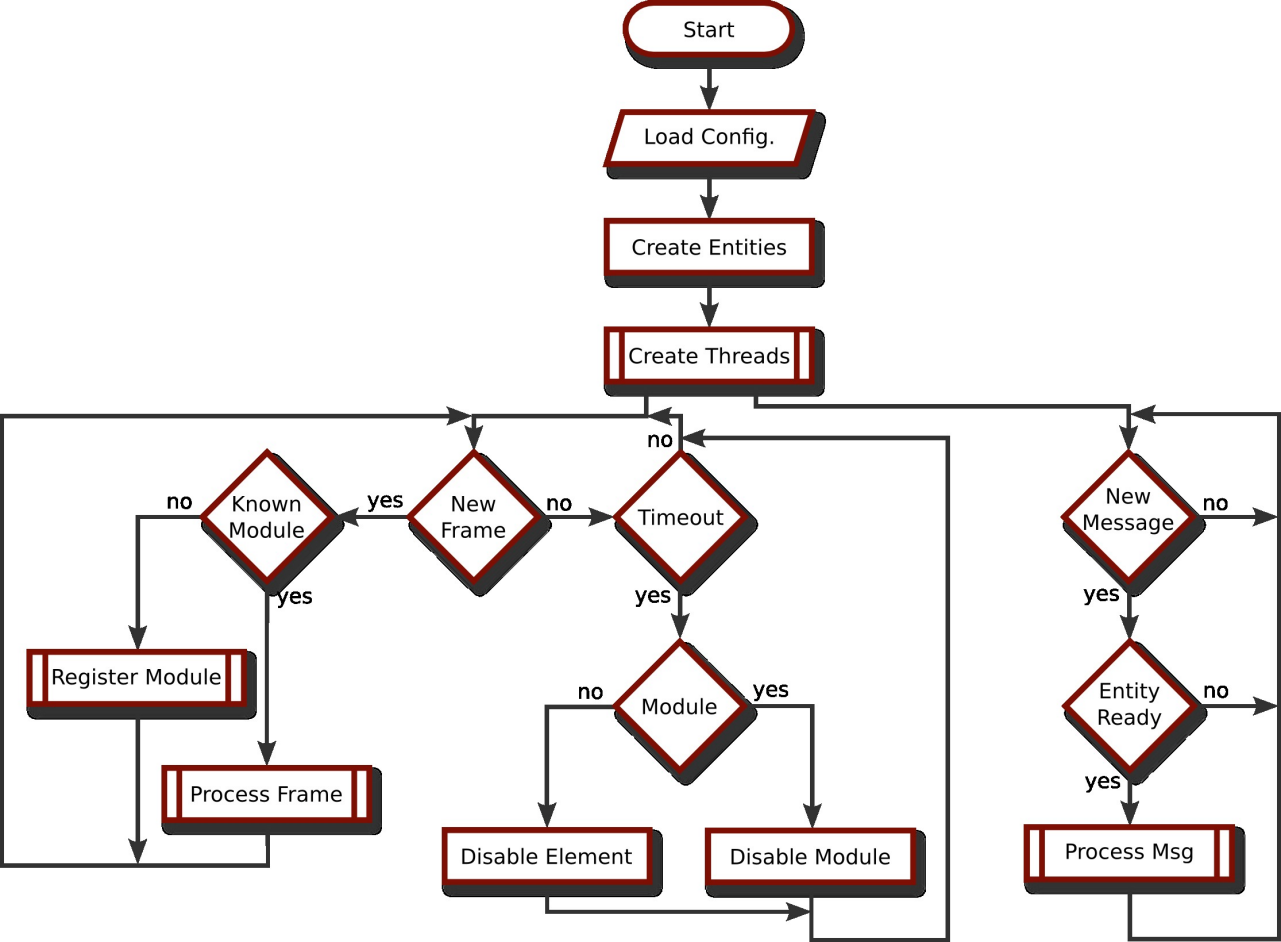
Communication flow chart.

The CAN interface is used to communicate with the distributed hardware nodes handling the connection, configuration and working mode of each distributed software element running into the slave modules. The RoboCAN server starts without any slave module registered and when a frame is received, it creates a new module instance, requesting information about what kind of software elements it contains. The server has two lists with the different drivers needed to manage the distributed components and agents. For each kind of distributed software element executed in the hardware layer, the server dynamically loads the appropriate driver, if it has not been loaded yet. Figure 6 shows the communication and driver loading procedures in the RoboCAN server.

Some of the distributed software elements have more than one working mode, and the driver is in charge of deciding which of them should be used in every moment. As an example, a PID agent can control a motor in position or in velocity, or a sonar sensor can work with different firing sequences to avoid cross-talk between sensors.

The distributed nodes can be disabled, avoiding the publication of data frames, even if they keep being attached to the bus and powered on. They can be disabled as a request from the master or due to an internal timeout. Timeouts are also important to detect on-the-fly disconnections and errors in any module, mainly hardware, but in some cases software errors can also be detected. If a distributed node is disabled, all the associated drivers are notified.

### 6.2. IPC Communications

RIDE architecture uses IPC to communicate the navigation modules [27]. Developed at Carnegie Mellon’s Robotics Institute, IPC provides a publication–subscription model for applications to exchange messages among modules via a central server process. Each application registers with the central server, and specifies what types of messages it publishes and what types it listens for. Any message sent to the central server is immediately forwarded to the subscribed processes.

The RoboCAN server makes use of the existing messages created in the architecture to exchange data between the control layer and the hardware servers. In Figure 5, the different interfaces (groups of messages) are represented as entities. The entities also define the relations between the high level IPC messages and the elements in the distributed hardware.

When starting, the server requests the system configuration to determine which entities must be used. For every needed entity, the system loads the appropriate dynamic loading library. These libraries are implementations of abstract entities and contain the hardware configuration, including the geometry dependencies. As an example, two different entity libraries are loaded to manage three different distributed modules that make up a differential robot (two for motor control and the other one for sonar and bumper sensors). One library implements the base entity and the other one implements the sonar entity. They also contain geometric parameters such as gear ratios for motors, PID gains, encoder resolutions, the firing sequences for the sonar and the bumpers positions.

## 7. Modules and Prototypes

**Figure 7:**
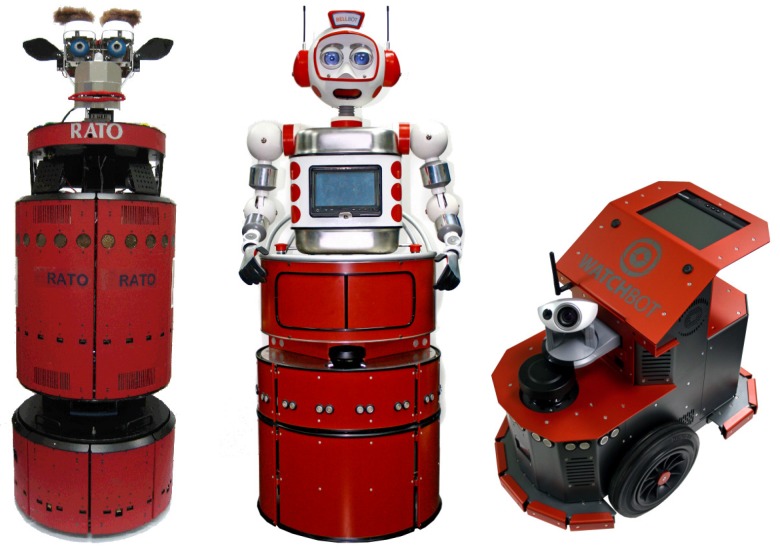
Robots with the RoboCAN solution installed (from left to right: Rato, BellBot and WatchBot).

The whole system described in the above sections has been used and tested in different robots and applications, Figure 7. In this section, we present the main software components and agents included in the RoboCAN integration architecture, as well as a detailed hardware analysis describing the different modules created.

**Figure 8:**
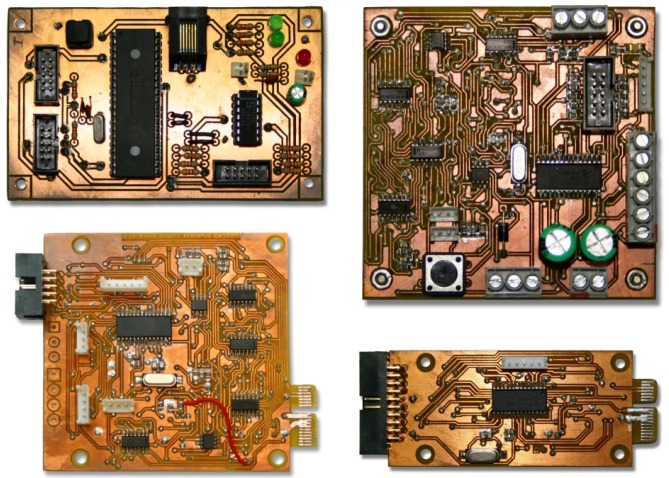
RoboCAN hardware modules developed under the three different generations. (**top-left**) Sonar and bumper controller in PDIP; (**top-right**) PID controller in SMT; (**bottom-left**) PID controller with PCI-E; (**bottom-right**) Sonar and bumper controller with PCI-E.

The concept was first applied to an old B21r robot from RWI, presented in [28]. The B21r robot has a set of hardware devices developed in PDIP (Plastic Dual In-Line Package) through-hole technology. Each of these modules was designed based on a specific PIC microcontroller to manage sensors, actuators, and also for the slave nodes to communicate with the RoboCAN server. For example, the first module created, depicted in Figure 8 (top-left), controls eight sonar sensors and eight bumpers with an 8-bit PIC18F458 MCU. Another example is a PID controller for a DC (Direct Current) motor with a quadrature encoder and bumper sensors based on a 16-bit dsPIC30F4011. In the end, the RoboCAN system was composed of three modules to handle the 24 sonar sensors and 24 bumpers on the top enclosure; two modules for the motor controllers and the mobile platform bumpers; one module to control the robot head and another module to handle the enclosure buttons and interface signals.

With the embedded software developed, proved and correctly tested in the modules already installed in the b21r robot, the manufacturing technology was changed to SMT (Surface Mount Technology) to create more compact hardware devices for a new robot design. This is a service robot to assist in hotels [29] known as BellBot. It has a synchronous base, a sonar sensors ring, bumpers, arms with servomotors, capacitive sensors for human interaction and a head also with capacitive sensors, RC (Radio Control) servos and lights. It resulted in a set of modules of three different types to manage all the sensors and actuators. Two modules for motor control and bumpers, another couple of twin modules for sonar control and the last one to manage RC servos, digital inputs, digital outputs and analog outputs. All the hardware is controlled with only five simple and low-cost modules, executing different components and also some agents, such as a Predictive PID for motor control [30] and a firing sequences for the sonar ring.

The module in Figure 8 (top-right) is a Predictive PID for motor control, which also includes components to manage the motor, the encoder and the bumper sensors. It is developed in SMT technology with a core unit model dsPIC30F4012. The other modules used in BellBot are based on the 8-bit PIC18F285 for the sonar ring and bumper sensors and a 16-bit dsPIC30F40 TQFP-44 (Thin Quad Flat Pack) for RC servo and signals.

To install and reuse the modules developed on different robots, a dimensional and electrical specification for board design was included. The electrical specification relies on the use of a single PCI-E (Peripheral Component Interconnect Express) connector for low power in different voltage levels (3.3 V, 5 V, 12 V, 24 V and 48 V), communication buses (two CAN, I2C, USB) and other signals (reset, external clock). The dimensional specification defines the size of every module or set of modules to be connected to a common main board. Figure 8 (bottom-left) shows the same module represented in Figure 8 (top-right) designed under the third generation dimensional and electrical specifications. Other modules, Figure 8 (bottom-right), implemented with this technology are the sonar controllers to be used in the WatchBot robot [31], a small differential robot for surveillance applications.

## 8. Protocol Analysis

In this section, a performance analysis of the proposed hardware integration architecture is presented. The analysis is focused on the field bus communications bandwidth usage and on the transmission efficiency, both providing quality measures of the RoboCAN communication protocol.

**Figure 9:**
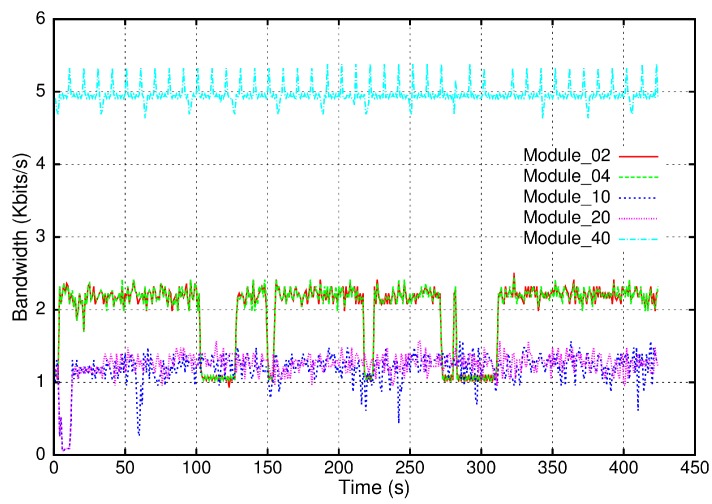
BellBot CAN bus bandwidth usage for each of its five distributed hardware modules.

The performance analysis is centered on the BellBot robot, a service mobile robot with human interaction capabilities. However, the proposed integration technology has already been applied to three different robots. As previously described, the integration and control hardware in this robot is a set of five distributed CAN nodes. Figure 9 shows the bandwidth usage for every distributed module. Module_02 and Module_04 are the base motion interfaces that contain velocity controllers based on Predictive PIDs and also the bumper sensors interface. Module_10 and Module_20 are the sonar ring controllers, each of them handling eight sonar sensors and eight bumpers. Module_40 contains a set of servos to control the robot head movement and the arms. It also contains input and output signals to handle the hands and face capacitive sensors, the eyes illumination and the locker doors. The bandwidth usage of every module depends on the number of elements managed (sensors and actuators) and also on the exchanged information. Module_40 controls a high number of different elements using more bandwidth than the other modules. On the other hand, the sonar modules handle less elements and also exchange data frames at lower frequencies.

In order to measure the bandwidth usage, all the traffic in the field bus was logged into a file containing the transmission time, the frame ID and transmitted data. For every distributed module, the bandwidth usage is represented in 1 s time windows, including the transmitted and the received frames.

The RoboCAN protocol performance were also compared with the CANOpen protocol to analyze its operational performance. A Schunk Powerball robot arm with six distributed CAN modules was used as a reference to compare the results. These CAN modules implement the CANOpen communications under the draft version DS402, with an 100 Hz data transmission frequency and a 20 Hz command reception frequency from a ROS node.

**Figure 10:**
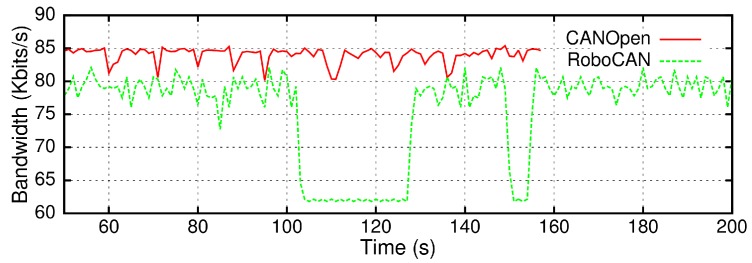
Bandwidth usage comparison between CANOpen and RoboCAN.

The operational specifications of BellBot, related to the motor controllers, are a 10 Hz publishing frequency and an 8 Hz receiving frequency. The results have been scaled to adapt the transmission and reception frequencies. Figure 10 shows that the RoboCAN protocol makes use of less bandwidth compared to the CANOpen DS402 specification. When the motors are moving, the RoboCAN bandwidth is about 6% less than the CANOpen and with the motors stopped it is about 25% less. The velocity command frames in CANOpen have a data length of 64 bits, while in RoboCAN the length is 56 bits, representing the 6% difference in bandwidth usage. The RoboCAN server does not send velocity frames while the motor is stopped. That explains the different bandwidth usage between the moving and the stopped states.

The CAN standard data frames under the A specification have an 11-bit ID and a total length between 47 and 111 bits, depending on the data transmitted. The maximum data transmission efficiency is limited to ξ=57.66%, obtained as the ratio between the transmitted payload and the standard frame length.

**Figure 11:**
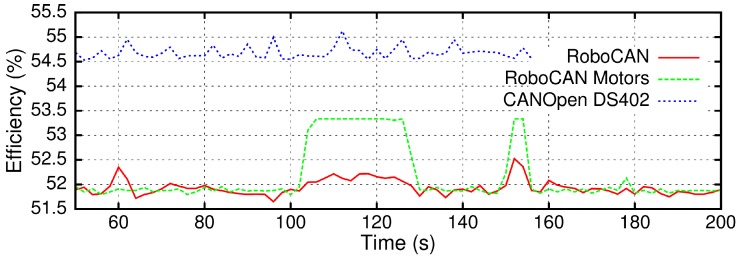
CANOpen DS402 and RoboCAN transmission efficiency.

Figure 11 shows the CANOpen DS402 and the RoboCAN transmission efficiency. In addition, as the DS402 is the motor control specific protocol, the RoboCAN motor controller data frames efficiency is also represented, isolated from the rest of the field bus traffic. The CANOpen protocol seems to be more efficient than the RoboCAN protocol because the data field of most frames is larger than in RoboCAN. However, as we have seen before, this better efficiency also leads to a larger bandwidth usage. The RoboCAN protocol is less efficient in reception than in transmission from the remote motor controllers. This is because of the data length difference between the command frames and the information frames, being more efficient when the controllers are stopped and not receiving new command frames.

## 9. Conclusions

The first conclusion that can be drawn from this research work is that RoboCAN is an efficient tool to generate distributed hardware modules, based on low-cost core units to integrate sensors, actuators and control devices in mobile robots architectures.

From the initial approach of distributed control units, the tool has evolved to the execution of software components and agents that are independent of the process core. The inclusion of hardware abstraction tables and interruption management procedures allows the reuse of software code in different families of microcontrollers. This provides a highly scalable system, allowing the creation of complex devices or applications, without modifying the approach used in the current implemented solution.

The RoboCAN tool was included as part of RIDE to facilitate the sensor and actuator integration, avoiding external dependencies and obtaining a better abstraction over the hardware layer. In this way, the control architecture can easily and homogeneously integrate new sensors, actuators and distributed control units without the need to rewrite software modules. Integrating the hardware layer into the control architecture facilitates the evolution of the whole system, keeping a better homogeneity in the information management and avoiding bulky and inefficient adaptation interfaces.

Regarding the creation of mobile robot applications, RoboCAN significantly reduces the development costs and the time required to adapt commercial sensors and actuators to the RIDE technology. This is, in part, because of the reuse of hardware modules and it is also due to the avoidance of the development of new software modules to integrate hardware devices. It is also important to note that the same communication framework can be used with other communication buses that are applied in mobile robots such as I2C [32]. However, as mentioned before, we consider that CAN provides a better performance for this kind of application.

The evaluation of this system shows that it presents a good performance regarding the communication parameters compared to other similar systems. Moreover, it is well suited for this kind of application, meeting the necessary requirements to integrate sensors, actuators and distributed control units in robotic architectures.

## Abbreviations

The following abbreviations are used in this manuscript:

CAN	Controller Area Network
RWI	Real World Interface
USB	Universal Serial Bus
RIDE	Robotics Integrated Development Environment
ROS	Robot Operating System
REU	Remote Engineering Unit
I2C	Inter-Integrated Circuit
DCF	Distributed Control Framework
RoboCAN	Robot CAN bus
SDS	Smart Distributed System
CAL	CAN Application Layer
CARMEN	The Carnegie Mellon Navigation Toolkit
GUI	Graphic User Interface
IPC	Inter Process Communication
MCU	Microcontroller
IMU	Inertial Measurement Unit
PDIP	Plastic Dual In-Line Package
PIC	Peripheral Interface Controller
DC	Direct Current
SMT	Surface Mount Technology
RC	Radio Control
TQFP	Thin Quad Flat Pack
PCI-E	Peripheral Component Interconnect Express
